# Diagnostic accuracy of self-collected menstrual blood for high-risk human papillomavirus testing for cervical intraepithelial neoplasia and cervical cancer: a systematic review and meta-analysis

**DOI:** 10.3389/fmicb.2026.1782917

**Published:** 2026-04-21

**Authors:** Xuechao Ji, Xuexia Ji, Shaomin Chen, Xibo Wang, Xueliang Liu

**Affiliations:** 1 Department of Gynecology, Weifang People’s Hospital, Shandong Second Medical University, Weifang, Shandong, China; 2 Department of Pharmacy, Weifang People’s Hospital, Shandong Second Medical University, Weifang, Shandong, China

**Keywords:** cervical intraepithelial neoplasia, cervical cancer, high risk papillomavirus, menstruation, self-collected samples

## Abstract

**Introduction:**

Human papillomavirus (HPV) test is the most commonly used method for cervical cancer screening, but the participation rates remain low in many countries. Self sampling menstrual blood (MB) high-risk HPV (hr-HPV) test may offer a solution to this issue. The aim of this meta-analysis was to examine the accuracy of MB HPV test in detecting cervical cancer and cervical intraepithelial neoplasia (CIN).

**Methods:**

Electronic databases (PubMed, Embase, and Cochrane) were searched for articles published through October 23, 2024, with the following search terms: (men strual blood OR menstruation) AND (human papillomavirus). Observational studies (e.g., cross-sectional studies, retrospective studies and prospective studies) that reported cervical histopathological biopsy outcomes in women undergoing self-sampling MB HPV testing were included. Data extraction was performed by two independent reviewers according to the PRISMA guidelines. Meta-analyses were conducted based on sensitivities and specificities and corresponding 95% Confidence intervals (CIs) using bivariate random-effects model and hierarchical summary receiver operating characteristic (HSROC) model. The risk of publication bias and heterogeneity were also assessed. Data were analyzed from October to November 2024.

**Results:**

Seven studies involving 1,672 adult women were included. The overall sensitivity and specificity of hrHPV testing from the MB samples for the diagnosis of CIN and cervical cancer were 0.96 (95% CI = 0.74-1.00, I^2^ = 86.14) and 0.53 (95% CI = 0.11-0.91, I^2^ = 98.24), respectively.

**Discussion:**

The evidence from this meta-analysis, which includes women with and without CIN and cervical cancer, shows the high sensitivity and low specificity of the MB hrHPV test. Further large-scale diagnostic studies that combine multiple testing modalities are recommended to support and validate the application of the MB hrHPV test.

**Systematic review registration:**

https://www.crd.york.ac.uk/PROSPERO/view/CRD42024605195, Identifier CRD42024605195.

## 1 Introduction

According to global cancer statistics 2022, cervical cancer remains the fourth leading cause of new cancer cases and cancer deaths among women. Over 660,000 women were diagnosed with cervical cancer, and more than 348,000 women died from the disease (Bray et al., 2024). More than 90% of cervical cancer was caused by persistent high-risk human papillomavirus (hrHPV) infection (de Sanjose et al., 2010). hrHPV testing is an effective method that can be used to detect HPV infection and cervical cancer. It is adopted as the primary screening tool for cervical cancer in many countries. World Health Organization (WHO), American Cancer Society (ACS), and the United States Preventive Services Task Force (USPSTF) have recommended using HPV DNA detection as the primary screening test in screening and treatment approaches (WHO, 2021; Fontham et al., 2020; Curry et al., 2018).

Although the HPV test has high accuracy in detecting cervical cancer and precancerous lesions (Melnikow et al., 2018), many women are still unwilling or unable to participate in cervical cancer screening programs provided by healthcare workers (Møen et al., 2020). Additionally, limited medical resources in low- and middle-income countries make it harder for women to access screening by healthcare workers. HPV self-sampling could help overcome these challenges. HPV self-sampling using vaginal or urine samples has shown high agreement with traditional clinical sampling for cervical HPV (Arbyn et al., 2018; Cho et al., 2022; Sahasrabuddhe et al., 2014). Menstrual blood (MB) flows from the uterine cavity and cervix into the vagina, containing exfoliated cells from the cervix. MB has been shown to correlate well with several common serum tests, such as hemoglobin A1C and thyrotropin (Naseri et al., 2023). Moreover, compared to slides, urine, and fluid-based cervico-vaginal samples, dried blood spots are easier to transport and do not require cold chain storage, significantly reducing the infrastructure and logistics costs of transporting samples to HPV testing laboratories (Naseri et al., 2022; Reed et al., 2024). Researchers have found that MB might be used to screen for cervical cancer (Fairley et al., 1994; Liberty et al., 2023). The use of menstrual pads for HPV testing offers a non-invasive alternative, alleviating concerns about invasive methods while saving time and improving convenience. Therefore, the MB hrHPV test could be a valuable tool for collecting samples from women who are unable or unwilling to participate in traditional cervical screening programs, serving as an effective supplement to current HPV detection methods.

Several studies have reported the sensitivity and specificity of the MB hrHPV test for detecting cervical lesions and hrHPV infection. However, no meta-analysis has been conducted on this topic. A systematic review included five studies on the MB hrHPV test, but the results were inconsistent (Chakravarti et al., 2022). This study aims to evaluate the accuracy of the MB hrHPV test in detecting cervical intraepithelial neoplasia (CIN) and cervical cancer.

## 2 Materials and methods

This study was registered with PROSPERO (CRD42024605195) and performed according to the standard Preferred Reporting Items for Systematic Reviews and Meta-Analyses (PRISMA) statement.

### 2.1 Search strategy and eligibility criteria

We conducted an online search for English-language literature in PubMed, Embase and the Cochrane databases from the construction date to Oct 23, 2024. The detailed search strategies are described in the Supplementary Figure S1 and Supplementary Table S1. Studies were deemed eligible for inclusion if they satisfied 3 key criteria: (1) the study assessed the clinical accuracy of hrHPV test on MB samples as an index test in women; (2) the reference was the presence of CIN or worse via histopathological biopsy (colposcopic cervical biopsy/punch biopsy/endocervical curettage/conization/hysterectomy); and (3) the study provided numbers of true positive, false positive, false negative, and true negative results, or this data were derived from the published results of studies.

### 2.2 Study selection, data extraction, and quality assessment

All titles and abstracts for relevant studies were reviewed. Two authors (XCJ and XXJ) had independently reviewed the full text for study selection, and data extraction. Information regarding the participants characteristics, study design, date of the MB collection (menstrual cycle day, MCD), sampling and transport devices, DNA extraction methods, hrHPV assays (detection platform, kit, target genotypes), cervical lesion threshold and gold standard were collected in a comprehensive table (Table 1). We accepted the cutoff proposed by the manufacturer to define HPV positivity by tests.

**Table 1:** Characteristics of studies included in the meta-analysis.

Study	Location	Study design	Population characteristics	Date of the menstrual blood collection	Sampling device	Preservative for blood transportation	DNA extraction	HPV DNA assay	Gold standard	Lesion thresholds
Tsang et al. (2024)	China	Cross-sectional study	Total: 6 6 women aged between 34 and 45 years with a pathological diagnosis of CIN 1, CIN 2, CIN 3 or HPV infection	MCD 2	Sanitary pad with a collection foam swab	Foam swab	QIAamp® DNA Blood Kit according to the dried blood spot protocol	PCR	Colposcopic cervical biopsy	≥CIN1
Zhang et al. (2021)	China	Cross-sectional study	Total: 120 120 women with hrHPV infection, average age 33.9 years	MCD 1	Sanitary pad	Sanitary pad	Tiangen dried blood spot DNA extraction kit	Target capture sequencing	Colposcopic cervical biopsy	≥CIN1
Wong et al. (2018)	China	Cross-sectional study	Total: 402 (a) 265 women with pathological diagnosis of CIN or HPV infection; (b) 137 normal sexually active women served as controls	NR	Sanitary pad	Sanitary pad	QIAamp DNA Mini Kit according to the dried blood spot protocol	PCR	Colposcopic cervical biopsy	≥CIN1
Budukh et al. (2018)	India	Cross-sectional study	Total: 557 Population A: 192 women provided the menstrual pad samples and of which 189 underwent HC2 testing; Population B: 367 women provided their menstrual pads and of which 365 samples were processed for DNA extraction by PCR method. HPV positive cases, 10% randomly selected HPV negative cases underwent HC2 testing	MCD 1	Homemade menstrual pad (old cloth)/ sanitary pad	Menstrual pad	QIAamp DNA Micro kit according to the dried blood spot protocol	PCR	Colposcopic cervical biopsy	≥CIN1
Lee et al. (2016)	Republic of Korea	Prospective exploratory pilot study	Total: 19 19 women with HSILs or HR-HPV infections, average age 35.05 ± 6.35 years	MCD 1	Sanitary pad with a filter	The pad filter	QIAamp DNA Mini kit or LaboPass Tissue Miniprep kit	PCR	Punch biopsy, endocervical curettage, conization or hysterectomy.	CIN2+/CIN3+
Wong et al. (2010)	China	Cross-sectional study	Total: 558 (a) 235 women with pathological diagnosis of CIN, and condyloma acuminatum (b) 323 sexually active normal subjects in the control group	NR	Sanitary pad	Sanitary pad	QIAamp DNA Mini kit according to the dried blood spot protocol	PCR	Colposcopic cervical biopsy	≥CIN1
Tong et al. (2003)	China	Cross-sectional study	Total: 10 10 women including seven with a pathological diagnosis of HPV infection, two with grade 1 CIN, and one with invasive squamous carcinoma of the cervix	NR	Sanitary pad	Sanitary pad	NR	PCR	Colposcopic cervical biopsy	≥CIN1

CIN, cervical intraepithelial neoplasia; HPV, human papillomavirus; MCD, menstrual cycle date; PCR, polymerase chain reaction; hr-HPV, high-risk human papillomavirus; NR, data not reported.

Two authors independently evaluated the quality of the included studies using the Quality Assessment of Diagnostic Accuracy Studies-2 (QUADAS-2) tool, which includes four domains: patient selection, index test, reference standard, and flow and timing. Each domain was assessed for risk of bias and applicability concerns. Disagreements between the authors were settled by consensus involving a third author if necessary.

### 2.3 Data analysis

The absolute sensitivity and specificity of the hrHPV test on MB samples were calculated using a bivariate model in the MIDAS module of STATA 17.0, a software designed for meta-analysis of diagnostic accuracy studies. Diagnostic accuracy metrics, including sensitivity, specificity, and diagnostic odds ratios (DORs), were reported as point estimates with 95% confidence intervals (CIs). The DOR was defined as the ratio of the odds of a positive test result in subjects with the disease to the odds of a positive result in subjects without the disease. The hierarchical summary receiver operating characteristic (HSROC) model was used to generate the summary ROC curve and calculate the pooled diagnostic accuracy.

Statistical heterogeneity across studies was assessed using I^2^ statistic and the Cochrane Q test, based on a random-effects model. Sensitivity analyses were conducted using a leave-one-out procedure to evaluate the robustness of the results. Likelihood ratios were used to assess the clinical utility of the test, and posterior probabilities, based on Bayes’ theorem, were calculated using Fagan’s nomogram.

Publication bias was examined using Deeks’ funnel plot asymmetry test for pooled absolute accuracy estimates. All statistical tests were two-sided, with significance set at *p* < 0.05. Analyses were conducted using STATA (version 17.0) and Review Manager (version 5.4).

## 3 Results

### 3.1 Study characteristics

A total of 23 studies from PubMed, 19 studies from Cochrane, and 10 studies from Embase were identified. No articles were found by scanning the reference lists of the identified studies. 47 references were remained after excluding duplicates. Seven studies including 1,672 adult, female participants were included in this review at the end. The flow of references through the selection process is shown in Supplementary Figure S1.

The patient populations of the included studies were women in China (*n* = 5) (Tsang et al., 2024; Wong et al., 2010; Wong et al., 2018; Zhang et al., 2021; Tong et al., 2003), India (1 study) (*n* = 1) and Republic of Korea (*n* = 1) (Lee et al., 2016). Most included studies were cross-sectional studies (*n* = 6). Only one study was a prospective exploratory pilot study. The timing of menstrual blood self-sampling differed substantially across included studies: samples were collected on menstrual cycle day 1 (MCD 1) in the studies by Lee, Budukh, and Zhang; on cycle day 2 (MCD 2) in the study by Tsang; whereas the exact cycle day was not specified (NR) in the studies by Wong et al. (2010, 2018) and Tong et al. (2003). The cervical lesion thresholds for diagnosis were unified as ≥CIN1 in 6 studies, and CIN2+/CIN3 + in 1 study (Lee et al., 2016). The summary of the population characteristics, study design, MCD of sampling, hrHPV assay details, lesion threshold and gold standard are available in the Table 1.

### 3.2 Quality assessment

**Figure 1:**
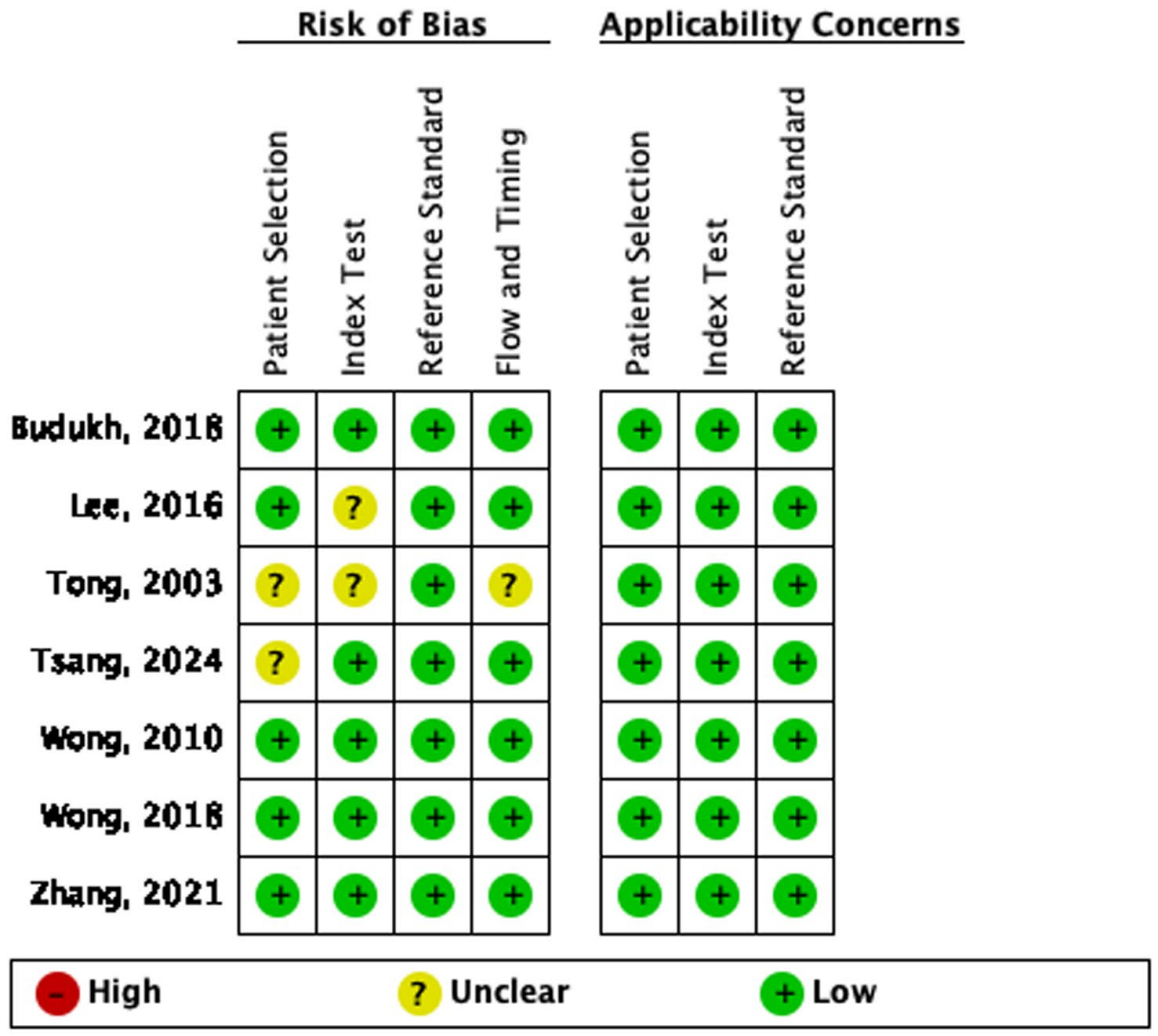
Risk of bias and applicability concerns summary: review authors’ judgments about each domain for each included study.

**Figure 2:**
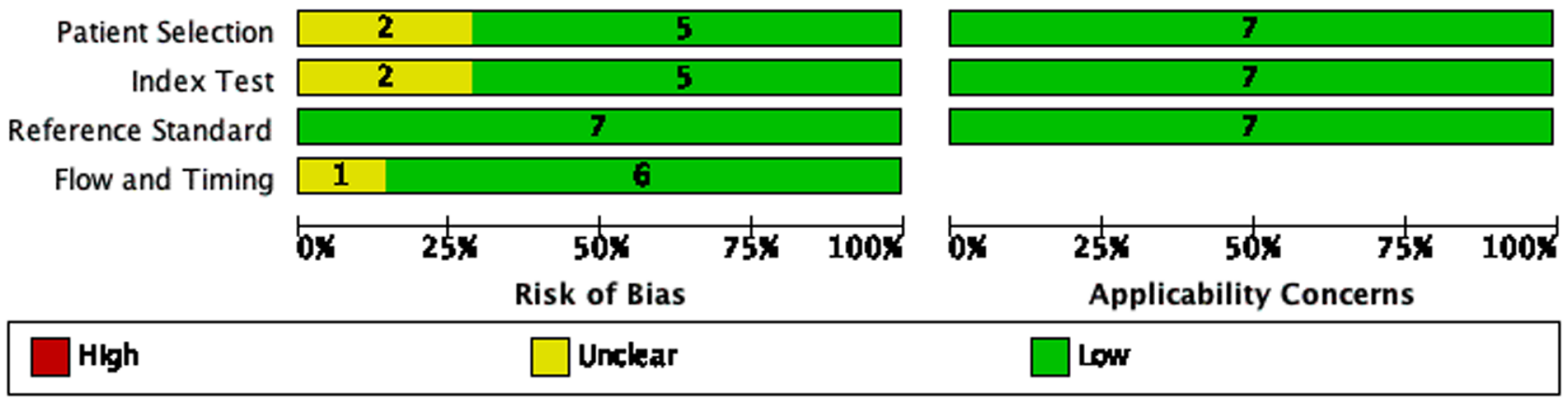
Risk of bias and applicability concerns graph: review authors’ judgments about each domain presented as percentages across included studies.

Figures 1, 2 show the QUADAS-2 assessment in the meta-analysis. The overall quality of studies appeared to be adequate: Patient selection: 5/7 (71%) studies had low risk of bias, 2/7 (29%) had unclear risk; all studies had low applicability concerns. Index test: 5/7 (71%) studies had low risk of bias, 2/7 (29%) had unclear risk; all studies had low applicability concerns. Reference standard: 7/7 (100%) studies had low risk of bias and low applicability concerns (all used histopathological biopsy as the gold standard). Flow and timing: 6/7 (86%) studies had low risk of bias, 1/7 (14%) had unclear risk; all studies had low applicability concerns.

All 7 studies showed low risks of bias in reference standard, which is the core domain for diagnostic accuracy studies and ensures the reliability of the included studies.

### 3.3 Estimates of the diagnostic accuracy of MB hrHPV test for detecting CIN

**Figure 3:**
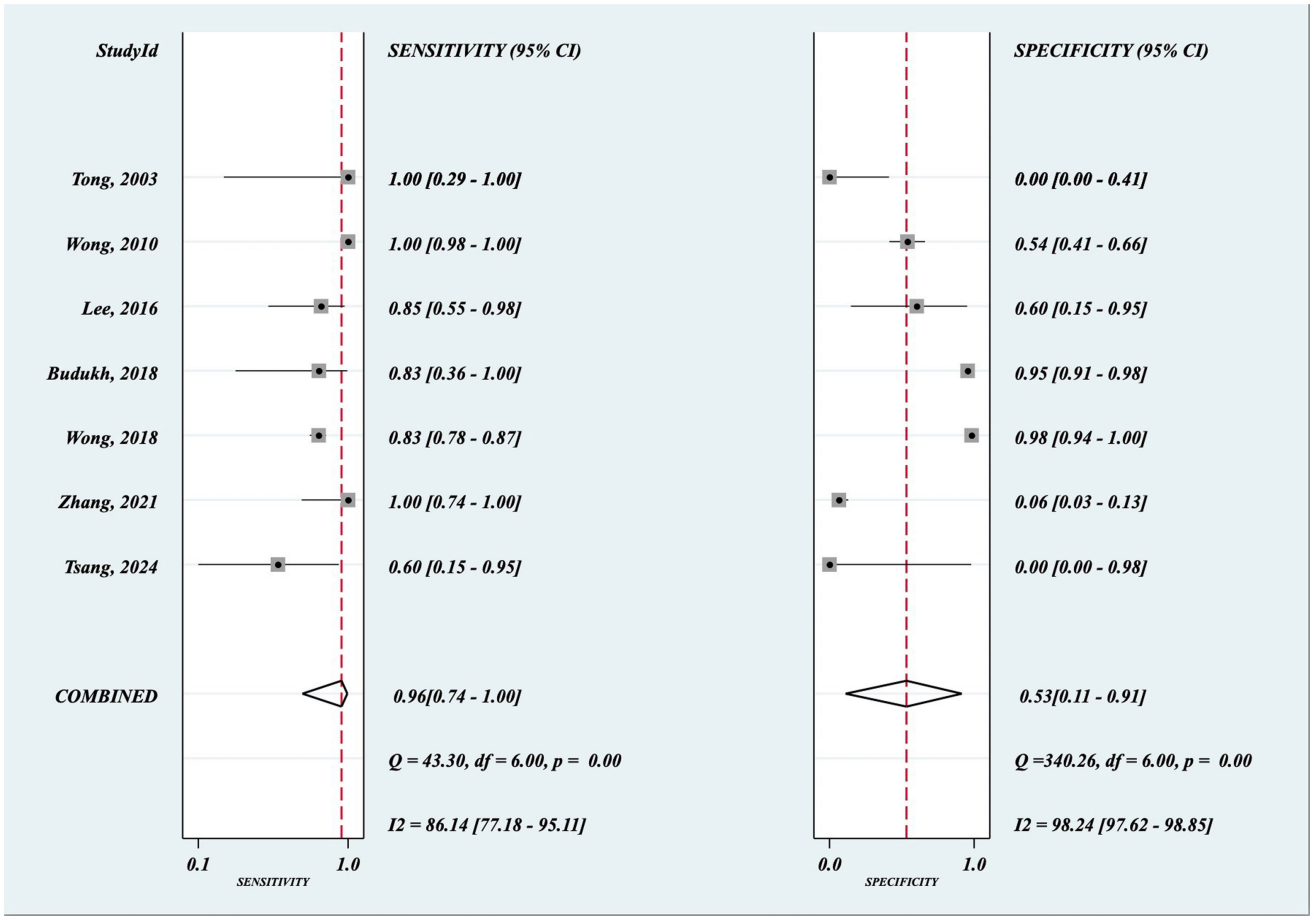
Forest plots of sensitivity and specificity for menstrual blood HPV test and using histopathological biopsy as a reference standard. MB, menstrual blood; HPV, human papillomavirus; TP, true positive; FP, false positive; FN, false negative; TN, true negative. Values between brackets are the 95% CIs of sensitivity and specificity.

**Figure 4:**
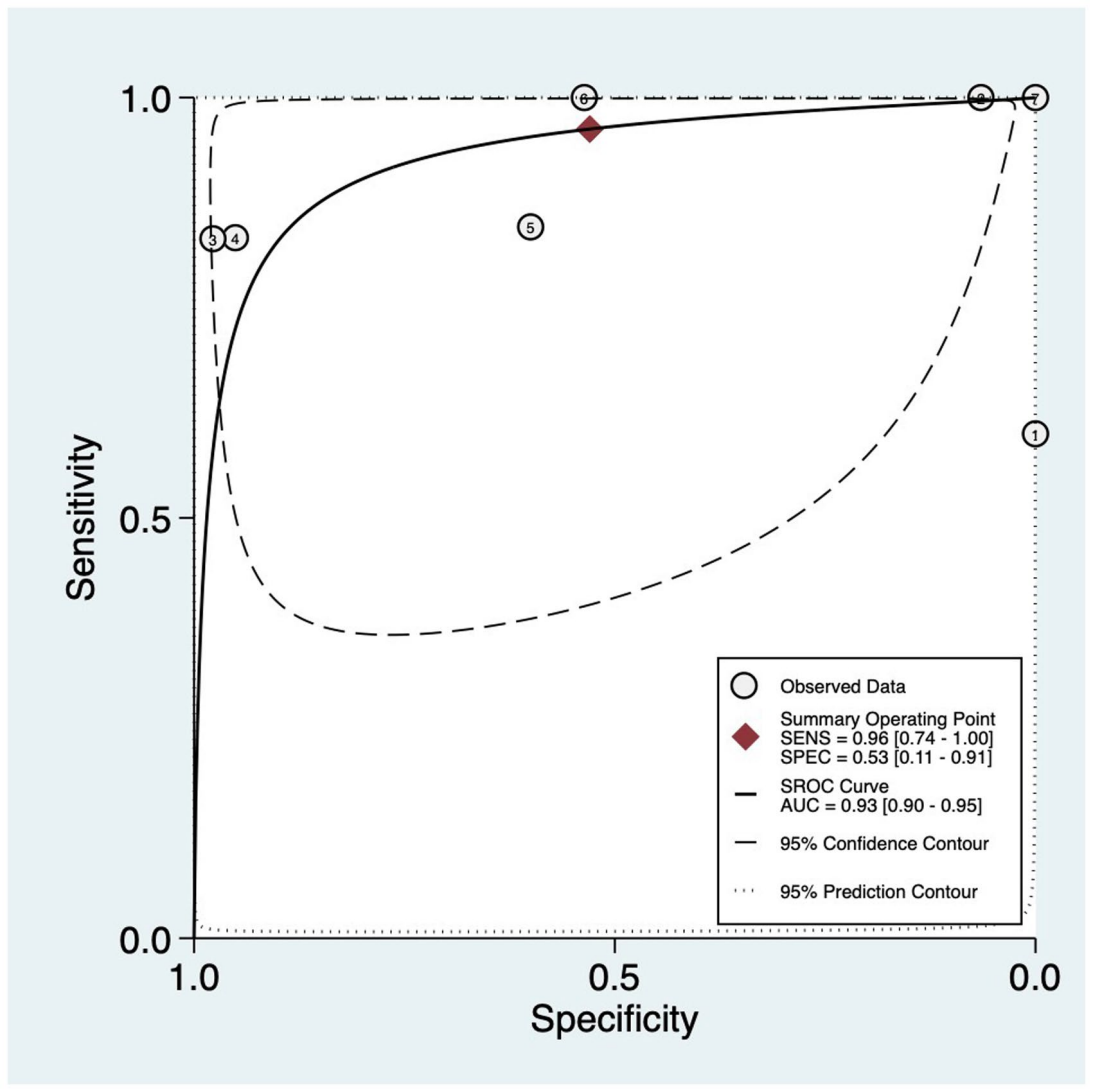
Summary plots of 7 studies investigating the diagnostic ability of MB HPV test to detect cervical intraepitelial neoplasia and cervical cancer. The solid circles correspond to the summary estimates of sensitivity and specificity and are shown with 95% confidence regions (dotted lines) and 95% prediction regions (dashed lines). MB, menstrual blood; HPV, human papillomavirus.

The sensitivity and specificity of each hrHPV test on MB samples is demonstrated in Figure 3, as well as the pooled sensitivity and specificity. The summary receiver operating characteristic (SROC) curve along with the summary point 95% confidence interval (CI) and prediction regions are illustrated in Figure 4.

The overall pooled sensitivity and specificity of MB hrHPV testing for the diagnosis of CIN and cervical cancer were 0.96 (95% CI = 0.74–1.00, I^2^ = 86.14) and 0.53 (95% CI = 0.11–0.91, I^2^ = 98.24), respectively. The high I^2^ values indicated severe statistical heterogeneity in both sensitivity and specificity across the included studies.

### 3.4 Investigations of heterogeneity

As shown in the forest plot (Figure 3) and I^2^ statistics, there was considerable high heterogeneity between the studies in terms of sensitivity (I^2^ = 86.14%) and specificity (I^2^ = 98.24%). The main potential sources of heterogeneity were identified as follows: (1) Timing of MB sampling: MCD 1, MCD 2 and unspecified sampling time across studies, leading to differences in cervical exfoliated cell content in MB samples. (2) hrHPV assay heterogeneity: Diverse detection platforms (ABI7500 real-time PCR, conventional PCR, QRT-PCR, HC2, Sanger sequencing) and target genotypes (from single HPV16 to 40 HPV types) across studies. (3) Sample size variation: Severe sample size differences [from 6 participants in Tsang et al. (2024) to 558 in Wong et al. (2010)], with 3 studies having sample size <20. (4) Study population differences: Populations from different countries (China, India, South Korea) with different hrHPV infection spectra and cervical lesion prevalence. Due to the limited number of studies included (*n* = 7), subgroup analysis and meta-regression with covariates to formally explore potential sources of heterogeneity were not feasible, and we stated this limitation in the Discussion section.

### 3.5 Sensitivity analyses

Supplementary Figure S2 showed the result of leave-one-out sensitivity analysis, which suggested the relatively stable results of the meta-analysis. No single study had a significant impact on the pooled sensitivity and specificity estimates, indicating that the overall diagnostic accuracy results of MB hrHPV testing were robust.

### 3.6 Clinical application value

Fagan’s nomogram was drawn to evaluate the clinical application value of the MB hrHPV test (Supplementary Figure S3). When the pre-test probability of CIN/cervical cancer was set at 50% (general high-risk population), the positive likelihood ratio (PLR) was 2, and the post-test probability was 67%. When the pretest probability was set at 50%, the negative likelihood ratio (NLR) was 0.07 and the post-test probability was 7%.

The low NLR (0.07) indicated that the MB hrHPV test had high negative predictive value: a negative test result could effectively rule out the presence of CIN/cervical cancer in women. The relatively low PLR (2) indicated that a positive test result needed to be confirmed by further examinations (e.g., colposcopy, vaginal self-sampling hrHPV test). These results show that the application of MB hrHPV test in the diagnosis of CIN has important clinical significance, especially for ruling out cervical lesions in women who refuse traditional screening.

### 3.7 Publication bias

Publication bias was not explored due to the insufficient number of studies included.

## 4 Discussion

Most of the studies included in this meta-analysis were hospital-based and cross-sectional. The results revealed that the pooled sensitivity and specificity of MB hrHPV testing for screening cervical intraepithelial neoplasia (CIN) and cervical cancer were 0.96 and 0.53, respectively. These findings suggest that while the MB hrHPV test has excellent high sensitivity, its specificity is relatively low. A high sensitivity indicates that the test can correctly identify most cases of CIN, whereas the low specificity suggests a higher rate of false positives, which could increase the burden of further clinical examinations (e.g., colposcopy and biopsy) and affect clinical diagnosis efficiency.

The main reason for the low specificity is the unified lesion threshold of ≥CIN1 in most included studies: the diagnosis of mild cervical lesions (CIN1) is more likely to produce false positive results, and if the threshold is raised to CIN2+/CIN3 + (high-grade lesions), the specificity of the test is expected to be significantly improved. In addition, the high heterogeneity of sampling timing and hrHPV assays also contributes to the low pooled specificity.

Self-collection methods, including MB, urine, and vaginal samples, represent emerging approaches for detecting CIN. However, the sensitivity and specificity of MB hrHPV detection are notably lower than those of the other two methods (Arbyn et al., 2018; Cho et al., 2022; Cho et al., 2019; Arbyn et al., 2023). Among the studies included in this review, polymerase chain reaction (PCR) was the most commonly used method for HPV detection. Other assays, such as Hybrid Capture 2, Cobas HPV, and APTIMA HPV, have shown variable performance in terms of sensitivity and specificity in previous studies, but these were not included in the MB hrHPV detection assays reviewed here (Cho et al., 2019; Goldstein et al., 2020; Chen et al., 2014; Chen et al., 2014). Furthermore, the transportation of sanitary pads from participants’ homes to the laboratory may also influence the test results, adding another potential source of variability (Asare et al., 2022). Therefore, combining the MB hrHPV test with other diagnostic methods (e.g., p16/Ki-67 dual staining) and optimizing transportation protocols (e.g., cold chain for fresh samples) may help improve diagnostic accuracy.

The heterogeneity across studies was high, with an I^2^ value of 86.14% for sensitivity and 98.24% for specificity, indicating significant variability in the results. Due to the limited number of included studies, we were unable to perform formal subgroup or meta-regression analyses to explore potential sources of this heterogeneity. Factors such as the timing of sample collection during the menstrual cycle, the type of MB collection devices, and the hrHPV assay platforms may contribute to the observed variability (Liu et al., 2013). Specifically, the sensitivity of MCD 1 sampling (77.8–94.2%) was significantly higher than that of MCD 2 sampling (66.7%), which is consistent with Okodo et al.’s (2022) theory that cervical exfoliated cells are washed by menstrual blood in the late menstrual cycle, leading to insufficient cells for hrHPV detection. Additionally, three studies in this review had small sample sizes (less than 20 participants), which may further explain the high heterogeneity.

The sensitivity analysis suggests that the main conclusions of this meta-analysis are stable, indicating that the findings hold up under different assumptions and adjustments. However, the small sample sizes in some studies may limit the generalizability of these results. The use of Fagan’s nomogram for visualizing posterior probabilities can help physicians optimize diagnostic decisions based on test outcomes, which could be especially useful in clinical practice to guide further testing or treatment plans: a negative MB hrHPV result can safely rule out CIN/cervical cancer, while a positive result needs further confirmation with vaginal self-sampling or colposcopy.

Patient acceptance of hrHPV self-sampling using MB was high, particularly among women who are reluctant to undergo traditional cervical screening (Wong et al., 2018; Hald et al., 2025). The comfort, privacy, convenience, and lower cost associated with MB self-sampling contributed to higher participation rates in screening. In low-resource settings, the MB hrHPV test has the potential to complement routine cervical swab screening. However, larger-scale, multi-center studies with larger sample sizes are needed to further evaluate the diagnostic effectiveness of MB hrHPV self-sampling in diverse populations.

In addition, further development of self-sampling tools and hrHPV detection platforms is crucial to improve test sensitivity and accuracy. Optimizing the sampling process to minimize errors and biases is also essential. Ensuring women’s privacy during self-sampling is a critical concern. In certain cultural contexts, MB self-sampling may face social taboos or cultural barriers, and overcoming these challenges to increase acceptance and participation remains an important consideration.

### 4.1 Study limitations

This meta-analysis has several limitations that should be acknowledged: (1) Only 7 studies were included, which is insufficient for a robust meta-analysis and makes it impossible to perform subgroup analysis and assess publication bias; (2) Severe high heterogeneity across studies due to sampling timing, hrHPV assays and sample size differences; (3) No head-to-head comparison with vaginal/urine self-sampling, which limits the evaluation of the clinical value of MB self-sampling; (4) Most studies used ≥CIN1 as the lesion threshold, leading to low pooled specificity; (5) The included studies are from only three countries (China, India, South Korea), which limits the generalizability of the results to other populations.

## Data Availability

The original contributions presented in the study are included in the article/Supplementary material, further inquiries can be directed to the corresponding authors.
