# Supplementary material for: Diagnostic accuracy of self-collected menstrual blood for high-risk human papillomavirus testing for cervical intraepithelial neoplasia and cervical cancer: a systematic review and meta-analysis
Source: Front Microbiol. 2026 Apr 21;17:1782917. doi: 10.3389/fmicb.2026.1782917 (PMC13142701; doi:10.3389/fmicb.2026.1782917)
Supplement: Supplementary file 1 [file Table_1.docx]

**Supplementary material 1**

**eTable 1: Detailed search strategy**

**Pubmed:**

((Human Papillomavirus DNA Tests[MeSH Terms]) OR (HPV DNA Tests[Title/Abstract]) OR (DNA Tests, HPV[Title/Abstract]) OR (Tests, HPV DNA[Title/Abstract]) OR (HPV DNA Tests[Title/Abstract]) OR (HPV[Title/Abstract]) OR (Human Papillomavirus[Title/Abstract])) AND ((menstruation[MeSH Terms]) OR (menstruation/blood[Title/Abstract]) OR (menstrual blood[Title/Abstract]))

**Cochrane:**

#1(menstruation):ti,ab,kw OR (menstruation blood):ti,ab,kw OR (menstrual blood):ti,ab,kw

#2 (Human Papillomavirus DNA Tests):ti,ab,kw OR (HPV DNA Tests):ti,ab,kw OR (DNA Tests, HPV):ti,ab,kw OR (Tests, HPV DNA):ti,ab,kw OR (Human Papillomavirus):ti,ab,kw

#1 AND #2

**Embase:**

#1 “menstruation”:ti,ab OR “menstruation blood”:ti,ab OR “menstrual blood”:ti,ab

#2 “Human Papillomavirus DNA Tests”:ti,ab OR “HPV DNA Tests”:ti,ab OR “ DNA Tests, HPV”:ti,ab OR “Tests, HPV DNA”:ti,ab OR “Human Papillomavirus”:ti,ab

#1 AND #2

**eFigure 1: Study flow diagram**

**
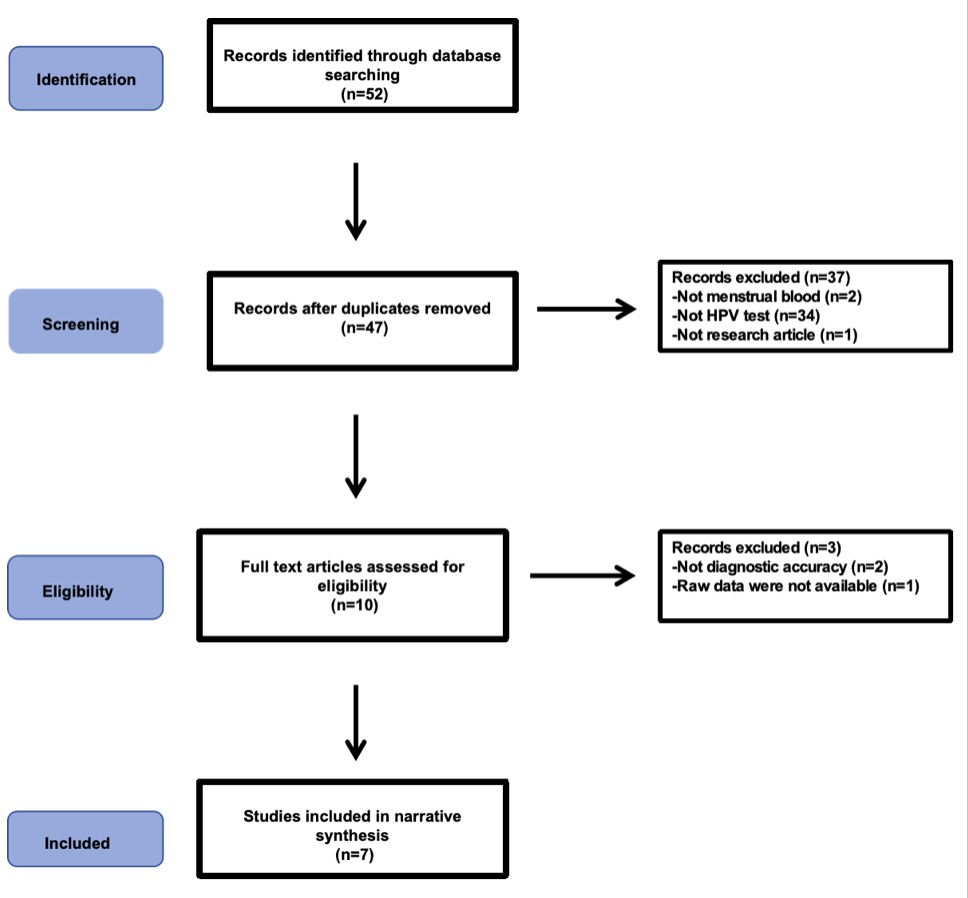
**

Abbreviations: HPV, human papillomavirus.

**eFigure 2: Sensitivity analyses in meta-analysis of MB HPV test for detecting CIN.**


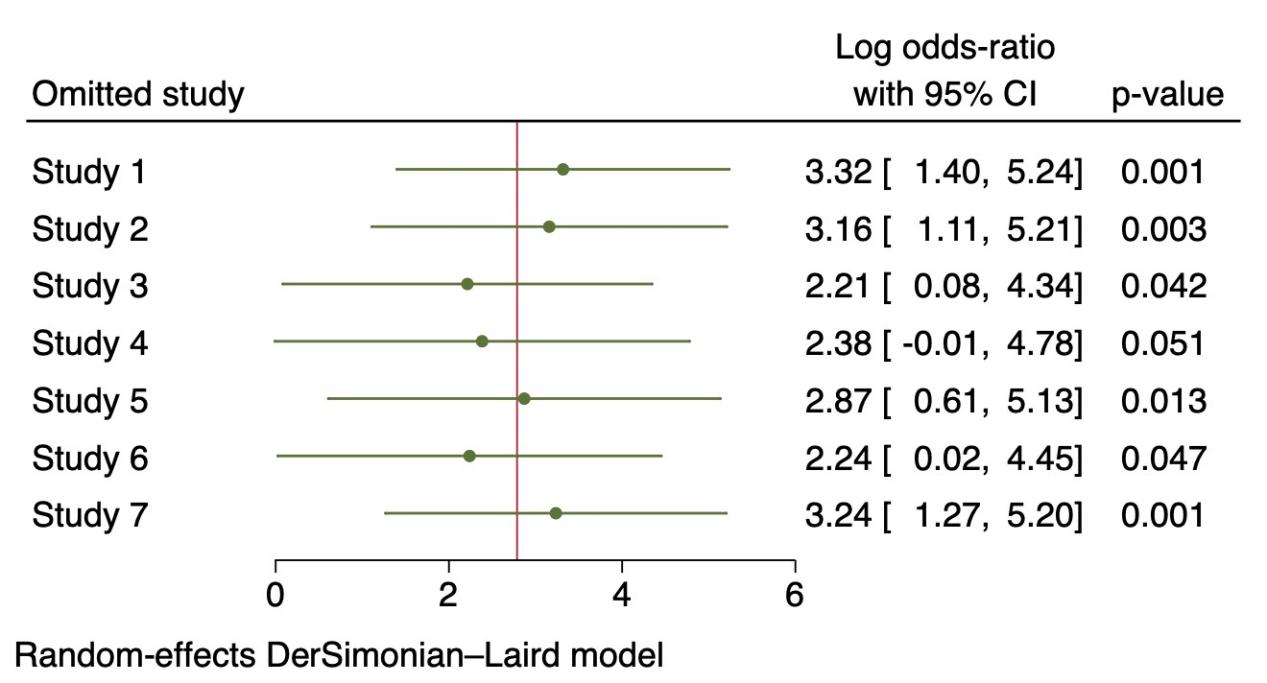


Abbreviations: MB, menstrual blood; HPV, human papillomavirus; CIN, cervical intraepithelial neoplasia.

Study 1: Tsang, 2024; Study 2: Zhang, 2021; Study 3: Wong, 2018; Study 4: Budukh, 2018; Study 5: Lee, 2016; Study 6: Wong, 2010; Study 7: Tong, 2003.

**eFigure 3: Fagan plot estimating how much the result of MB HPV test changes the probability of a CIN condition, considering a given pre-test probability.**

**
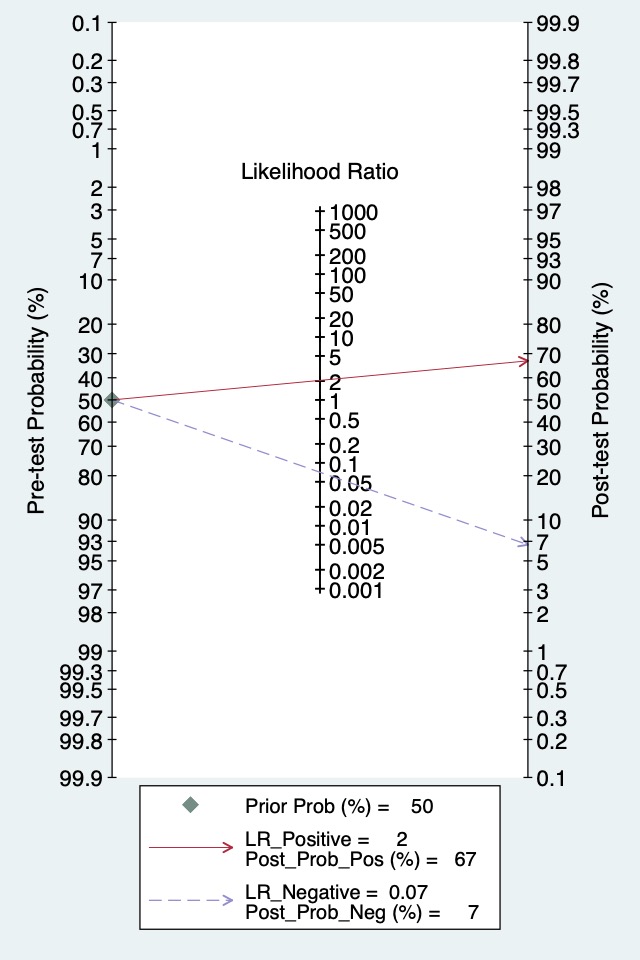
**

Abbreviations: MB, menstrual blood; HPV, human papillomavirus; CIN, cervical intraepithelial neoplasia.
